# Pig meat production in the European Union-27: current status, challenges, and future trends

**DOI:** 10.5713/ab.23.0496

**Published:** 2024-04-01

**Authors:** G. G. Mateos, N. L. Corrales, G. Talegón, L. Aguirre

**Affiliations:** 1 Departamento de Producción Agraria, ETSIAAB, Universidad Politécnica de Madrid, 28040 Madrid, Spain

**Keywords:** African Swine Disease, European Union-27, Feed and Meat Production, Pig Meat Quality, World Pork Trade

## Abstract

The main objective of this study was to present data on the current situation and future trends of pig meat production in the European Union-27 (EU). Pig production has played an important social and economic role for centuries in many states of the EU. In 2022, pig meat production in the EU reached 23 M tons, which represented 21% of total production worldwide. The two key reasons that justify such amount of pork produced, are the acceptance and high consumption of the meat by the local population and the high quality of the meat produced which facilitated pork export. However, current data show a reduction in pork production for the last three years, as a consequence of a series of events that include i) problems with the chain of ingredients supply, ii) uncontrolled increase in African Swine Fever (ASF) outbreaks, iii) fast recovery of pig production in China, iv) increasing concerns by the rural population on the high cost to meet future requirements of the EU legislation on farm management, environmental sustainability and animal welfare, v) increased cost of all inputs involved in pig production and vi) limited interest of the new farmer generation to work on the pig sector. Consequently, pork production is expected to decrease in the EU for the next years, although sales will be maintained at a relative high level because pork is the meat preferred by local consumers in most EU countries. In order to maintain the favourable position of the pork industry in the near future, strategies to implement include: i) maintain the quality of the meat destinated to export markets, ii) improve the control of outbreaks of ASF and other swine diseases, iii) implementation of technological innovations to improve working conditions making more attractive to work in the pork sector of the food chain to the new generation of farmers and workers.

## INTRODUCTION

Pigs were first domesticated in the Near East around 8,500 BC and brought by agriculturists to Northern Europe by 4,500 BC. But confusingly, the DNA of modern European pigs seems to indicate that the current pig population derives also from the domestication of European wild boars [1]. Later on, pigs were brought to South Europe and introduced by the Phoenicians from the Eastern Mediterranean cost (area of Lebanon) into the Iberian Peninsula (Spain and Portugal) where they interbred with local wild boars. Pig production systems have changed rapidly for the last 200 years, passing from small size, extensive systems, with a key role in the subsistence of the peasants and the maintenance of rural social structure, to very intensive, industrialized systems, designed to feed a growing urban population. In fact, pigs adapt quickly to changes in the production system, from extensive production, such as the Iberian pig in Spain and Portugal and the Mangalica pig in Hungary and Romania, to sophisticated intensive operations based on highly selected pig breeds, such as Large White, Landrace, Duroc and Pietrain, among others.

## WORLD MEAT PRODUCTION

**Figure 1:**
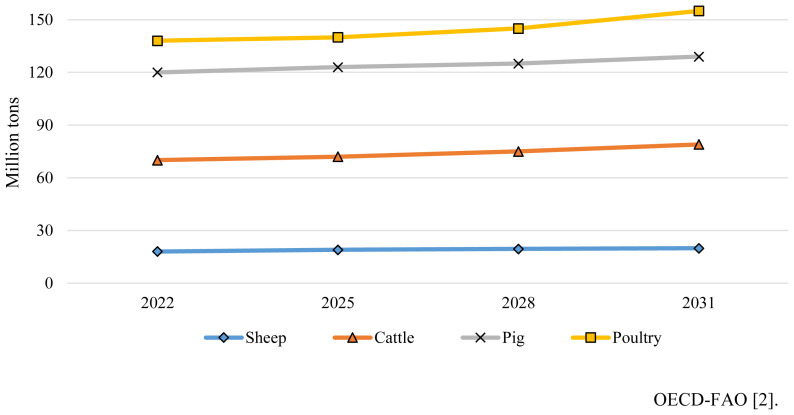
Projected meat production volume by species (World trends).

Global meat production has increased steadily for the last decades, reaching 362.6 Mt in 2022. In the last years, world meat production was distorted due to the COVID-19 pandemic and to losses linked to the occurrence of diseases such as foot and mouth disease in beef cattle, highly pathogenic Avian influenza in poultry and African Swine Fever (ASF) in pigs, which had a deep economic impact in all these three meat sectors. In this respect, unexpected outbreaks of these diseases led in numerous occasions to the loss of substantial volumes of exports and local sales of meat, affecting the economy of the entire meat sector. A marginal increase of 0.4%, is forecasted for the next year mostly because of the 1.3% increase of poultry meat (Table 1). Global production of pork, however, is expected to drop by 0.5% for the same period, mostly a consequence of the decline in the European Union-27 (EU). Based on increasing profitability, as meat prices rebound from the post-COVID-19 situation and the expected decline in inflation and feed cost, meat consumption is expected to reach 382 Mt worldwide in 2032 [2]. The increase in meat consumption (Figure 1) will be led by poultry (1.3% to 156 Mt), followed by pork (0.6% to 129 Mt), beef and veal (0.9% to 78 Mt) and sheep (1.3% to 19 Mt). By 2032, worldwide production of pork, poultry, beef and sheep meats are expected to grow by 11%, 15%, 10%, and 15%, respectively with poultry meat accounting for 41% of the animal protein source consumed from all meat sources [2].

Historical data on worldwide production of pig meat and swine stock, provided by USDA (2023), are shown in Tables 2 and 3. China has been traditionally the main pork producer country in the world, with approximately 50% to 55% of all the production, followed by the EU and the USA with 22% and 11%, respectively. At present time, the EU is the main consumer of pig meat (kg/capita) with 41.1 kg, followed by China, USA, Russia and Brazil with 37.2, 29.5, 24.1, and 17.7 kg, respectively [3]. From 2012 to 2021, pork production increased in the EU and some American countries, such as USA, Brazil and Canada. In 2022, exports were led by the EU with 38% of the total, followed by USA, Canada and Brazil with 26%, 13%, and 12%, respectively. On the other hand, China (22%), Japan (15%), Mexico (13%), United Kingdom (8%), and South Korea (7%) were the main importers (Table 4). After 2022, however, the main changes have been a decrease in pork production in the EU, caused by the implementation in previous years of measures on social distances, because of the incidence of COVID-19 which resulted in shortages of labor in slaughterhouses and meat processing and packaging plants, forcing often to the shutdown of key operations in export countries, compensated by an increase in China (Table 2). From 2002 to 2020, the export of pig products (on carcass weight bases) from the EU to extra-EU countries increase from 2.50 to 6.38 M tons, (a 255% increase) with China, United Kingdom and Japan leading the pork trade (Table 5). However, a clear reduction in these 3 countries and in total exports have been observed for the last three years. In contrast, pork meat imports of the EU from extra EU countries has been negligible (Table 6) with UK and Switzerland as main suppliers. According to USDA (2023) [4], pork exports in the last decade was led by the EU but decreased by 12% from 2019 to 2023, a decline that is expected to continue in the next future (Table 4). Recent, pig meat forecasts [3] indicate that Asia, with an increase of 11.9%, will lead pork production from 2022 to 2031 (Table 7). Increases expected around the world vary from 7% to 8% for America to 19.6% for Africa whereas a decrease of 5.1% is expected for Europe. In this respect, the increase in pork production in Asia and North America, might compensate for the decrease in Europe [3,5]. The expected increase in production in the Asian Pacific countries will benefit of the closing of small backyard holdings, compensated in volume by an increase in large scale commercial enterprises with better biosecurity measures. This change in pig production system, together with the potential availability and use of “adequate” vaccination programs against ASF [6], might help to control the spread of the disease in the near future.

## PIG PRODUCTION STRUCTURE IN THE EUROPEAN UNION-27

In 2022, the EU had a sizeable livestock population, with 134 M pigs (−5% compared with 2021), 75 M bovines (−1%), 59 M ovines (−2%), and 11 M goats (−3%) [7]. Swine production, with 9% of the total agricultural output and 35% of total EU meat output in 2018 [8], was the major contributor to the agriculture economy of the EU. Geographically, the main pig production basin extends from Denmark to Belgium which in 2014 accounted for over 30% of all the EU sows [9] and with three major countries (Spain, Germany and France), accounting for over 50% of the total pork production. At present, the specific regions in which pig production is concentrated, are Jutland in Denmark, North Brabant in The Netherlands, West Flanders in Belgium, Lower Saxony and Rhine-Westphalia in Germany, Brittany in France, Lombardy in Italy, Wielkopolskie in Poland and the Northwest and the Mediterranean regions (Cataluña, Aragón and Murcia) in Spain. In 2016 [7], the countries with greater number of pigs per 100 habitants were Denmark (n = 215), Netherlands (n = 70), Spain (n = 63), and Belgium (n = 54). In 2022, the statistics show a decline in this ratio in most countries, with Denmark (n = 194), Spain (n = 71), and the Netherlands (n = 60) having the greater ratios, whereas Poland (n = 26), Germany (n = 25), France (n = 18), and Italy (n = 15) showed the lowest numbers among the key countries (Table 8).

Pig farming in the EU corresponds to one of these four general systems: i) traditional small scale backyard production, ii) industrialized, large scale, intensive indoor production, iii) extensive outdoor operations, with pigs reared under free range conditions and direct consumption of natural resources, and iv) organic production, of limited economic importance at present time (less than 2% for the EU) but growing in the last years [10]. A description of the characteristics of each system is presented below. Pork production systems, farm size and pig management vary widely between and within member states, from small traditional holdings keeping just one or two pigs, such as in Romania and other Eastern EU countries, to conventional intensive indoor production, with industrial installations housing thousands of pigs, such as in Denmark and Netherlands [11].

1) Backyard farming is still the more predominant traditional system of keeping pigs in many EU states in which the main objective is to supply an economical source of animal protein to the rural population. For the last decades, pig farms became larger in size but fewer in number in the EU, and pork production shifted from backyard to the intensive indoor confinement system, with greater potential to raise pigs in a more cost-efficient manner. In fact, the number of farms holding pigs in the EU, has decreased over the years from 3.8 M in 2005 to just under 2.2 M in 2015, with more than half of the farms, at the end of this period, placed in Romania. Backyard production has been heavily affected by the ASF epidemic. As an example, in 2022, 87% of the total outbreaks of ASF (n = 327) in the EU in 2022 occurred in Romania [12] which reduced further the future interest and importance of this system. In fact, in 2023, less than 2% of the pigs of the EU were kept under the backyard system, with an even lower share in the key EU countries. However, close to 98% of the farms had less than 10 pigs, although many small pig farms in many states (e.g., Romania, Poland, Check Republic, and Hungary) are closing due to the deterioration of the economic situation and lack of profitability of their business.

2) Pig production in big units under intensive conditions and high biosecurity measures standards is the predominant system in Western Europe, with a continuous increase in all countries for the last 30 years. Overall, over 75% of the EU pigs are in large commercial holdings. The new industrial holdings are market-oriented enterprises with focus on satisfying the needs of the pork processing industry, the growing urban population and the export markets.

3) The production of pigs under extensive conditions (organic and non-organic) with animals reared outdoor, was important in the last decades of the 20th century in many regions of Europe, including the Iberian Peninsula, Hungary, Bulgaria and the Mediterranean islands. In this traditional system local pig breeds are used, resulting in low cost of rearing but reduced productivity. In the last decades, the number of pigs reared under this system decreased in all countries, except for the Iberian pigs in Spain and Portugal.

Originally, the Iberian pigs were reared outdoor, under total extensive conditions during their whole lives. However, as a consequence of the high incidence of ASF outbreaks (started in Portugal) in the mid of the 20th century, and the decrease of the price of animal fats, Iberian pig numbers decreased exponentially until the end of the century, and then increase steadily for the first decades of the 21st century. Currently, the meat of these pigs are an important component of high value sector of the Spanish pork production chain [13]. At present time, the system has diversified into different types, according to management, feeding program, production system and type and quality of the final products. In this respect, Iberian pigs might be reared i) under free range conditions and fed on acorns, pasture and other natural resources, exclusively for the whole fattening period (90 to 115 kg body weight [BW] to slaughter), ii) reared indoor and fed compound feeds similar in composition to those of the traditional white pigs, or iii) intermediate management systems (Table 9). Under any of the 3 systems, the sows must be certified as being of the Iberian breed but the males, depending on the quality required, can be “Iberian” (more expensive) or Duroc (higher productivity) breeds. The pigs are slaughtered with a minimum of 150 to 160 kg BW and 10 to 14 months of age, depending on the selected type of pig and as indicated previously, both males and females are castrated. Most of the carcasses of the Iberian pigs are destined to the dry-cured industry, as added value products. As an example, at the supermarkets the price of dry-cured ham in 2023, varies from 10 to 15 €/kg for the traditional Large White×Landrace breeds, cured for six months, to over 200 €/kg for the highest quality Iberian pigs in which the drying cured process might last for 24 months or more.

4) Organic pig husbandry systems can basically be divided into three major categories: i) outdoor housing, which might include access to pasture under free-range conditions for part of their lives, ii) indoor housing with access to a concrete outside run for part of their lives, and iii) mixed housing [10,14]. Organic production concentrates in Germany, Denmark and other North-Central European countries. In all cases the housing and management practices, compulsory for the production of organic pigs, vary considerably among countries but require always that the animals can have, someway, access to outdoor. In spite of the fast growth in recent years, organic pig production in the EU remains below 2% in in comparison with 5% to 7% for the main ruminant species.

The EU pig sector does not have the level of integration seen in the poultry sector, with some exceptions, such as Denmark and Spain. In Denmark, most pigs are controlled in many aspects, by few companies, with Danish Crown playing a relevant position. Management, including the control of emergent diseases such as *Salmonella* spp and ASF, type of production, fatteners selling price, as well as sustainability and animal welfare issues, are centralized and tightly control, following common criteria. In Spain, a large part of the pork production chain is vertically controlled by family owned companies. The integrator provides the pigs (often also the sows), the feeds, the veterinarian services and their own standards for farm management and each type of production. Farmers are contracted exclusively to keep and take care of the fatteners or breeding sows in their farms. In the last decades, slaughterhouses and meat processing companies, in most key EU countries, such as Denmark, Netherlands, Germany and Spain, have increased in volume, showing also a high level of integration.

In 2022, the average population density in the EU was of 109 inhabitants per km2, a number than varied from 18 to 1,657 among member states [15]. Among the key pork producer countries, the highest human density corresponds to the Netherlands (429), Belgium (378), and Germany (233) and the lowest to Spain (94) and Romania (84). Pig density (animals/km^2^) and the ratio of number of pigs per person (Table 8) are very high in Denmark (267 and 1.94), Netherlands (258 and 0.60) and Spain (67 and 0.71) whereas the lowest figures are for Italy (29 and 0.15) and France (20 and 0.18). In 2015, the average size of the farms was of 3,500 heads in Denmark, 2,500 in Netherlands and approximately 1,050 in Germany, with more than 90% of the pigs kept on farms with at least 1,000 animals. As expected, based on economy of scales, farm size increased to 4,700, 3,400, and 1,300 heads in 2018 in the three countries mentioned. The difference in farm size among EU countries, depends at a great extent, on the local legislation, with tighter restrictions for increasing farm size, reported in the most developed countries with the greatest density in human population. A special case is Romania, a country in which the pig industry is highly polarized, with an increase in large intensive farms, which supply 85% of all the meat available on the market, most run by foreign companies at one end, but still a high number of backyard pigs producing meat for the family and local neighbours at the other end.

In the EU, pigs are usually slaughtered with over 110 kg BW and less than six months of age with males physically castrated at birth [16]. However, in some countries (e.g., Spain) a large proportion of pigs, are not castrated. In addition, an increased proportion of pigs are reared up to 125 kg BW and seven months of age (e.g., Spain) or to 160 to 170 kg BW and nine months of age (e.g., Parma ham pigs, Italy), with males castrated at birth. Also, Iberian pigs (Spain and Portugal) reared under free range conditions, are slaughtered at 150 to 180 kg BW and 10 to 12 months of age and both, males (at birth) and females (at two months of age) are castrated (Table 9). The raising of pigs for meat are organized in different ways with pig husbandry being more or less sophisticated, depending on the size of the farm, the needs of the farmers and the production system selected. Small farms combine usually sows and fatteners, with little emphasis for improvements in genetic or management practices. Modern operations, however, might rear all pigs (sows, piglets and fatteners) in the same facilities or use farms in which sows and piglet up to 20/25 kg BW, are separated from the growing-fattening pigs, with the first unit needing greater care and more specialized personnel.

## PIG CENSUS AND TRENDS

From 2012 to 2021, the number of pigs slaughtered and of tons of pork produced increased in the EU (Tables 10 and 11) but an opposite trend was observed thereafter (Tables 12 and 13). On carcass weight bases, pork production from 2012 to 2021 increased from 22.7 to 23.6 Mt, and self-sufficiency from 111% to 125%, indicative that the increase was driven primarily by exports (2.2 to 4.7 Mt) to third countries (Table 11). In fact, from 2012 to 2022, pork consumption did not change much but a 0.4% year decrease, from current 32.0 to 31.1 kg/capita, is expected from 2022 to 2032. For the last years pig census showed a record high of 145.9 M in December 2020 and a record low of 134.3 M two years later. In fact, pig inventory decreased from of 142.6 M in 2016 to 134.3 M in 2022 (Table 12), with a further 6% decrease expected for 2023 [17]. In fact, the number of pigs slaughtered in the first seven months of 2023 is of only 116.8 M [18]. However, for the last decade, changes in pig and pork numbers were not uniform among countries, with the highest decrease pigs slaughtered (2015 to 2021) reported for Germany (−14%), a reduction that was compensated by an increase of 27% in Spain (Table 10). Furthermore, from 2021 to 2022 pig inventory decreased by more than 10% in Germany and Denmark and close to 6% in France and Poland, whereas in Spain for the same period, was of only 0.9% (Table 12). In terms of pork production, the largest decrease in 2022 vs 2021 took place in Germany, Denmark and The Netherlands (236, 202, and 144 Mt), representing declines of 9%, 21%, and 15%, respectively [19]. Moreover, from January to July 2023, pork production decreased by 8% (12.8 to 11.9 Mt) as compared with same period in 2022. All this information agrees with data of the EU Commission [20] confirming a reduction in sow census from 10.1 M in 2021 to 9.8 M in 2022 (Table 14) with a further decrease to less than 9.5 M sows, expected by the end of 2023 [3]. The reduction in sow numbers (also due in part of to better productivity) expected, might reach 700.000 heads from 2013 to 2023. Based on current information, pork production of the EU had a sharp decrease for the last three years with an extra decline of 6% expected in 2023 vs 2022.

## DIAGNOSIS AND FUTURE OF PIG MEAT PRODUCTION IN THE EUROPEAN UNION-27. MAJOR CHALLENGES

Pork production has an important socio-economical role in many countries of the EU, particularly Spain, Germany, France, Denmark and The Netherlands (Table 12). The future of this meat sector depends on many factors of which the most important are i) future increases in pig production in Asia which will reduce exports by third countries, ii) increased unrest of the current economic and political situation worldwide, iii) uncontrolled new breaks of ASF and other diseases such as classical swine fever, porcine reproductive and respiratory syndrome (PRRS), actinobacillus pleuropneumoniae, circovirus, proliferative enteropathy (Lawsonia intracellularis) and Streptococcus suis, which will affect pig productivity, iv) per capita pork consumption that have decreased from 47.1 kg in 2015 to 41.1 kg in 2022, with further decreases because of inflation and the consumers trend to reduce meat intake for health or animal welfare issues [21], v) distortions of the energy and pork markets caused by inflation rate, which increases cost and reduces margins, vi) reduced availability of feed ingredients, labor and energy, and vii) poor understanding by the farmers of the key criteria of consumer demands for pork meat and on the legislation pressure on food safety, sustainability and animal welfare.

### Impact of pork production in Asia

Asia produced 63.9 M tons (carcass weight) of pork meat in 2022, with an increase of 7.5 M tons expected for 2031 (Table 7). For the first eight months of 2023, the export capacity of the EU fell in most countries comparing with data for the same months in 2022 and a further decrease is forecasted for next years [19]. The fall in export to third countries was the highest for China but in percentages was larger for Philippines (−42%) followed by South Korea (−34%), Japan (−20%), and China (−16%) [3].

### Impact of African Swine Fever on pork trade

African Swine Fever is a deadly viral, infectious disease affecting both wild boars and domestic pigs in many parts the world. It can be transmitted through direct animal contact, infected material or contaminated feed. There are not efficient prevention methods at present time and the only way to control the disease is through the elimination of infected pigs and the strict control of movements and contacts of personnel, pigs and pork products. However, the wild boar population is protected in many EU countries from hunting, and the free movement of the animals, within and between countries, can not be controlled in an efficient way, making difficult to think in a fast eradication of the disease [11].

African Swine Fever was first described in Kenia in 1921 and spread rapidly through several sub-Saharan countries. The virus was detected in Portugal in 1957 and moved to Spain in 1960, through wild boars and free-range domestic pig populations between the two countries. The disease was largely eradicated in the EU in the 1980s and 1990s, except in Sardinia (Italy) where it remained endemic. The first outbreaks of ASF in the EU in this century, appeared in the Baltic States and then, in Poland in 2014, creating an unexpected animal health crisis worldwide. The disease spread to other EU countries and a first outbreak was detected in Germany in wild boars in 2020. In January 2022, the ASF jumped from Sardinia, in which the disease was endemic, to the North of Italy. From January 2020 to September 2022, a total of 3,703 outbreaks in domestic swine and 20,518 in wild boars were detected in Europe [6,22]. Since the first occurrence in the EU in 2014, 2022 was the first year in which a reduction of outbreaks in wild boars was reported (from 12,076 cases in 2021 to 7,282 in 2022, representing a decrease of 40%). Poland with 2,152 and Germany, with 1,628 cases were the most affected countries.

In 2023, the ASF virus is still out of control, with a sharp increase in outbreaks, occurring in January affecting domestic pigs of the Balkan States (e.g., Bosnia and Herzegovina and Croatia). From January 2022 to July 2023 the AFS outbreaks in Europe were of 2,082 in domestic pigs and 8,626 in wild boars as reported by the WOAH [23]. For domestic pigs, the biggest number of cases (not individual pigs) reported for the EU were in the Balkan region including Romania (616), Serbia (590), Bosnia Herzegovina (432), and Bulgaria (432). By August 2023, the outbreaks detected in domestic pigs in this part of Europe were 312 in Croatia, 266 in Bosnia and Herzegovina, 292 in Romania and 239 in Serbia [19]. As per October 13th 2023, the total number of ASF outbreaks across Europe stood at 6,505, according to the European Commission s system. To date, 20 countries have registered one or more outbreaks through the system in 2023. For comparison, there were a total of 7,442 outbreaks of ASF in wild boars across 15 European countries in the whole of 2022 [17].

In 2018, a first ASF outbreak took place in China and spread rapidly through the continent (18 countries affected in the region), with Philippines, South Korea, Vietnam and China being the most affected in the last four years. At present time, the number of outbreaks among wild and domestic pigs appear to be declining in Asia, especially in China, in which ASF looks under control. Over the last decade, ASF had a dramatic impact on the pig sector, affecting more than 55 countries. It remains a threat for the future of the pork production industry worldwide.

The ASF outbreaks that affected China and the neighbour countries in the past, created an opportunity for the EU market, with Germany and Spain taken advantage of the situation. However, the posterior outbreaks in wild boars in Germany in 2020, reduced to a minimum the exports of this leading country, leaving Spain (currently exporting over 50% of its own pork production) as the leader for the Asian market. As a consequence, the demand and price for pig meat products in the third quarter of 2022 had a sharp increase in the EU However, the number of pigs ready for slaughter decreased on the first semester of 2023 creating an over capacity of the existing slaughterhouses and an increase, out of control, of pig meat price. The situation was non-sustainable, with slaughterhouses under pressure because of limited production and heavy economical losses, which ended in the closing of pork facilities in Germany, Denmark and other European countries.

### Distortion of the feed ingredient and pork markets

Volatility is always a problem in the trade market, and together with inflation, has been the main problem for the pork industry for the last three years. In 2021/2022, the expected decrease in local consumption and in pig meat exports, together with the increase in feed and energy cost, forced farmers to reduce their pig and sows inventory which led to a decrease in supply (Tables 14 and 15) and a concomitant increase in pork price in 2023. Pig meat prices (€/kg) in Germany, the second biggest pig producer of the EU, varied from 1.99 in 2021 to 1.65 in 2022 and back to 1.98 in 2022. Because the high prices for pork meat, in summer 2022 (over 2.00 €/kg BW in most countries, including Germany and Spain) the European pig producers were very optimistic and in fact, from April to September 2023 selling price reached values never shown before in Germany, with an outstanding maximum of 2.62 €/kg on July 19th, 2023 [3]. Similar situation occurred in most EU countries with prices in this period over 2.20 €/kg for pork meat and over 90 € for piglets with 20 kg BW. However, beginning fall of 2023, the trend changed with a sharp decrease in prices in many EU states (e.g., 1.70 to 1.60 €/kg in Spain) while production cost was maintained high, a situation that is not expected to change in the near future [19]. On week 49 of 2023, the average price of pig meat (100 kg carcass weight) in the EU-27 was of 212.4 €, an increase of 33% compared with the same week of the last year. In fact, the range of values per 100 kg carcass weight varied from 191 € in France to 261 € in Belgium, two neighbour countries. In the case of the piglets, the cost per unit was of 74 €, almost 30% higher than the price one year before [15]. In fact, in the last quarter of 2023, pork meat price in Spain was below 1.67 €/kg for fatteners, 0.91 €/kg for old sows, and 55 € for 20 kg BW piglets (still convenient, competitive prices).

The reduction in pork offer, together with the high local price of pork, affected the possibilities for exports in 2023, a position that will be occupied, at least in part, by the USA and Brazil (Table 4). For the last months of 2023, the disruption of the market, caused by the volatility in ingredients and meat prices worldwide, and the recovery of pork production in China, has caused a reduction in pork price in the EU (below 1.7 €/kg in many states, although still above 1.90 €/kg in Germany), a decrease which is not expected to recover in 2024 [24].

### Compound feed industry in the European Union-27

Global compound feed production in 2022 is estimated in 1,266 Mt (Table 15), with Europe representing 21% of the total [25]. According to this report, China with 261 Mt is the main producer, followed by the USA with 240 Mt, with Spain (31 Mt) and Germany (24 Mt) within the 10 major pork producer countries in the world (Table 16). The industrial feed production for domestic animals in the EU in this year was of 146 Mt [26], with Spain (25.8 Mt), Germany (21.9 Mt) and France (19.2 Mt) as leaders in the EU (Table 17). Pigs, with 51% market share led feed production in 2022, with poultry (48.9%) and ruminants (≈48%) being close. Spain was leader in feed production for pigs (25%) and cattle (22%) while France maintains its leading position for poultry (16%).

From 2021 to 2022, the production of industrial compound feed decreased by more than five M tons in the EU [27], a decrease that affected all sectors (e.g., −6.7% for pigs and −3.2% for poultry) and that is forecasted to continue in 2023 and 2024 (Table 17). The 10% decrease in pig feed production expected from 2023 to 2021, will affect especially to Denmark (20.7%), Germany (13.9%), Spain (9.0%), and France (8.4%). As indicated for pork production, the main market drivers, that justify a reduction in feed production in 2023, are the outbreaks of Avian Influenza and ASF, input cost, squeezing farm margins, reduced pork consumption (increases in living cost and in the percentage of flexi-vegetarian consumers), distortion of the traditional markets for ingredients (e.g., cereals and protein sources from East Europe), economy uncertainty and increased concerns on the future of all agricultural activities (e.g., “green and animal welfare” legislation).

Animal feed is the most important cost factor in meat production and represented in 2021, 62% of the cost value for poultry, 40% for pigs, and 14% for cattle [27]. Next to cost, the availability of ingredients plays an important role in the production process. The role of feed formulators, consists in producing feeds that fulfil the nutritional requirements of the animals, supporting best performance, while minimizing the cost. To achieve this objective, a mixture of feed materials is chosen in the most efficient way to reduce meat production cost. The number of feed mills has been decreased in most of the key countries of the EU with a concomitant increase in size [26]. The new feed mills are more efficient with the use of new technology, resulting in better feed quality and improved sustainability, which help to meet the objectives of the EU on animal production.

In 2021, animal feeds in the EU were based primarily on cereals (76.5% of total compound feed production), cakes and protein meals (39.2% mostly soybean meal, rapeseed meal, sunflower meal and peas), coproducts (18.0% from the food and bioethanol industry) and 2.6% of oils and fats (Table 18). Feed material used in feed formulation, including cereals, pulses and co-products from the food and bioethanol industries are primarily of EU origin and the deficiency in cereals is of only 10% [27]. The proportion of cereals (grains and their co-products) in the diets has remained stable for the last 10 years. The EU uses 50.1 Mt of oilseeds of which 56% is soybean meal or soybeans to be crushed locally (Table 19). In total, the EU imports 21 Mt of oilseed products, of which 16.4 Mt are soy products imported from America. Over 80% of all soybeans imported is used by five EU members: Netherlands, Spain, Germany, Netherlands, and Italy (Tables 19 and 20). Thus, the EU depends heavily on protein sources for animal feeding, a situation which might create problems of supply in case of international conflicts or future legislation against deforestation. There is a clear trend to reduce the percentage of oilseeds in the diet, a decrease that is expected to continue in the next future, because of the greater availability of industrial amino acids and other additives (e.g., enzymes) and the need to decrease environmental problems by reducing the protein contents of the diets and better control of ingredient imports. In this respect, the EU imported 28.2 Mt of soybeans meal equivalent (soybeans and soybean meals) in 2021 from which 30%, mostly of South America origin, came from areas with “deforestation risk” [27]. In 2021, the EU used 50.1 Mt of oilseeds feeds [28] from which, 56% were soybeans, 26% rapeseeds, 12% sunflower, and 3% palm kernel (Table 20). Overall, 77% of total feed proteins used are produced within the EU but the EU is mostly dependent (72%) on the import of ingredients with high rate of crude protein (more than 30%), especially soybean meal (97% imported). However, it is not easy to envisage which steps to take to reduce imports from third countries, although soybean production in the EU has nearly doubled in the last seven years and continues its upward trend, with Italy, France, and Romania as main producers [28].

### Understanding legislation pressure and consumer demands

Pig production under intensive production systems, rises numerous issues, linked in particular to environmental pollution, animal welfare and undesirable climate changes, which might affect feeding and management practices and pork production and thereby, it needs to be addressed by the legislators [8]. The final objective of the EU in relation to the pig industry is to produce more sustainable products with social value and economically feasible. The three main pillars of the EU strategy in relation to support pig production, include i) common organization of the markets, ii) rural development funding, and iii) legislation on environmental protection, organic production and pig welfare.

All steps of the pig production sector are regulated by a number of legislative acts [11], with relevance in the areas of food safety, public and animal health, environmental protection and animal welfare issues (e.g., General Food Law Regulation [EC, No 178/2002], Directive 2010/75/EU on Industrial Emissions, Council Directive 98/58/EC on pig welfare, and European Food Safety Authority [EFSA] control, etc). In this respect, the European Green Deal [29] was a response of the leaders of the EU to the current challenges of climate change and environmental degradation. One of the building blocks of this document, was the “Farm to Fork” strategy, published in May 2020. The strategy recognized the need to reduce the dependency of the EU on pesticides, fertilizers and antimicrobials and had, as main objectives, to improve animal welfare, increase organic farming and revers biodiversity loss.

Areas of concern of pig production with effects on pork cost include i) food safety for humans (e.g., ban of ZnO at pharmacological level and the use of in feed antibiotics), ii) animal welfare at the farm (e.g., weaning age, routine mutilation: tail docking and castration, use of sow crates, rearing density), iii) reduction and control of the levels of N, P, and trace elements in feeds iv) ban to use ingredients related to deforestation processes (e.g., soybean and palm derived products), v) transport conditions of the pigs from the farm to the slaughterhouse (e.g., trip length), and vi) protection of the environment, with a reduction in the number of pigs and limits to increase farm size in high density areas, which affects economy of scale, with negative effects on farm economy.

The key domains which promote positive welfare in pigs, are in addition to be free of pain, maintain a good health status plus having water and feed as needed give the animals opportunities for foraging, play, nest building and improve pig-human interactions [30]. The EU regulations prohibit routine tail docking, widely used in most countries to prevent tail biting and castration. Tail docking was banned in 1994, but problems for the enforcement of the ban in most of the EU states are widespread [30]. The main purpose of surgical castration in pigs is to avoid boar taint in the case of males and to prevent unwanted reproduction in females, a practice that has been under severe scrutiny for many years because of welfare concerns [16]. Surveys conducted in 24 countries from 2017 to 2019 indicate that 66% of the male pigs were castrated in the EU, in most cases without receiving any pain relief [10]. As a result of public concerns on pig castration, the immune castration approach might be a practical alternative in many cases [31].

According to the European Environmental Agency, agriculture was responsible for 94% of the ammonia emissions in the EU in 2015, with a tendency to increase in the last 10 years. Consequently, a major challenge facing the pig sector is the need to eliminate the negative impact on the environment when pigs are reared in zones under pollution pressure. Main concerns are related to water and air pollution but also odors and noises causing local disturbances needs to be controlled while maintaining at the same time the profitability of the farm systems.

## SWINE PRODUCTION PERSPECTIVES IN THE EUROPE UNION-27: FOCUSING ON KEY COUNTRIES

Pig census in the EU is concentrated (68% of total pork production) in five key countries: Denmark, The Netherlands, France, Germany and Spain (Table 12), each of them with their own drawbacks and advantages. The main downsides impeding an increase in this important segment of the EU economy, are indicated below:

1) Difficulties to control the spread of diseases, particularly ASF, which affects pig production and pork exports.
2) Increased pressure by consumers and legislators on food safety, sustainability and animal welfare issues, which will increase further pig production cost, with a concomitant reduction in competitiveness as compared with other countries.
3) Distorted markets affecting the availability and use of feed ingredients and pork selling prices, ending in problems in all the channels of the trade chain.
4) High density of human population in areas typically strong in pig production, which results in conflicts and incompatibilities between humans and pigs. A reduction in the number and size of the pig farms, which might occur in the most mature and developed members of the EU, will reduce pork production and eliminate the benefits of the economy of scale.
5) Increase percentage of flexitarian, vegetarian and vegan consumers, willing to reduce meat intake, with greater incidence in the young population of the most developed EU countries. An increase in transparent information to customers will be needed [32].
6) Farmer succession at risk because of the limited interest in pig production of the new generation.

On the other hand, the EU has a series of competitive advantages for pork production as shown below:

i) Leader in consumption of pig meat. Preference by the consumers for local meat over other protein sources.
ii) Social need to produce food at home for convenience and safety reasons (local production, higher confidence, extra marketing benefits and reduced risk of breaks in the supply chain).
iii) Good image of the quality of the EU pork (safe, uniform products, convenient and consistent supply) which facilitates exports to third countries.
iv) Excellent technology and fast implementation of know-how and new management practices as required for modern pig production.
v) Optimal situation of the feed mills (pig production close to ports or agricultural areas producing cereals) characterized, in general, for adequate size and excellent technology.
vi) Well organized markets on each of the key, individual countries, with a clear move towards integrated systems.
vii) Adequate health programs to control the spread of existing diseases (ASF, classical swine fever, PRRS, circovirus, ileitis by Lawsonia intracellularis, Streptococcus suis, etc.)

A brief discussion of the main characteristics of the pig and pork production sector in the five key countries of the EU is presented below.

### Denmark

Denmark is a relatively small country with a population of 5.8 M inhabitants and 13.2 M pigs slaughtered in 2021 which means they produce annually more than two pigs per habitant. Denmark is the country of the world with most pigs per capita, in which pig population (13.4 M pigs in 2020) was steady for long but suffering a continuous decrease in the last years to 11.5 M in 2022. Even more, for the first quarter of 2023, the number of pigs dropped by 20%, with a census figure that is the lowest since 1998.

Most of the Danish production of pig meat is intended for exports which impacts gravely the environment, the climate and the way of leaving of the local communities. Moreover, even though 80 % of the Danish agricultural land is used to produce feeds, with 150 Ha of land per pig farm, the amount produced is not enough and consequently, the industry depends heavily on imported crops, namely soybeans. The high density of inhabitants and pigs, together with the high cost of production (labor, energy, infrastructures, and tight legislation) makes difficult to think in a future increase in pig meat production. In fact, the number of farms has decreased from 27,300 in 1990 to less than 2,400 in 2022, in spite of the size of the farms (90% having at least 2,000 animals). Moreover, inflation has made increasingly difficult to cover the cost of pig meat production and as a result, some farms have closed, while others have sold their pigs for export. All these factors, together with the turmoils of the political situation, has resulted in a severe reduction in the number of pigs available for slaughter (a reduction of 20% from September 2023 to 2022 [15]) which has obliged to close some of the facilities and slaughterhouses, reducing further the amount of pork available in the country.

On the other hand, Denmark is considered as a benchmark of the swine industry worldwide, with an excellent image within the swine sector, which facilitates exports of piglets (e.g., Germany and Poland), breeders (e.g., Danbred sows) and pig meat all over the world. As an example, in 2021, Danbred sows produced as an average, 33.9 piglets/sow/yr [33] and the number of piglets produced by the top five Danbred farms was of 41.2 piglets (an increase of 4.3 piglet with respect to the 36.9 piglet recorded in 2017). The top 25% Danish enterprises produced 15.6 weaned piglets per farrowing, resulting in 36 piglets with an average BW of 6.2 kg weaned per year [34]. In addition, Danish pig production has been considered for long as a leader on sanitary practices, with adequate programs in relation to the control of key diseases (e.g., ASF, PRRS, and Salmonellosis) as well as in practices on animal welfare and environmental issues.

### The Netherlands

Pig production has been an important economic activity in The Netherlands and the country has been for long a leader in pig production and pork exports, with Spain, Germany, Poland and Rumania as the main buyers [3]. In 1996, the country had as many pigs as inhabitants and pig production came under pressure because of a large outbreak of classical swine fever that affected numerous farms and increased concerns of the population on environmental pollution issues. The situation led to a national legislation to control the growth of the pig sector in the country. A system (“pig rights”) was introduced in which the number of pigs in the country was fixed at around 12 M [35] and move many farmers to produce piglets and fattener pigs for exports. As per December 2022, pig census was of 10.7 M [7], a clear descend from the 11.9 M reported for the same month in 2016 (Table 12). At present, the ratio of pigs per inhabitant is of 0.60 (17.8 M people and 258 pigs/km^2^) still one of the highest in the EU (Table 8).

The future of pig production in the country is penalized by the high density of pigs in densely populated areas, the high cost of agricultural land and the potential problems with contamination because of the geography of the country, with one third of the land below sea level. In this respect, by 2030, at least 74% of the N-sensitive nature in protected nature areas, must have a healthy N level [36]. For the last 15 years the swine sector changed towards the increase in size of the farms and the production of piglets (up to ≈25 kg BW) destined to the export market in detriment of farms dedicated to pig fattening. In fact, in January 2023, the numbers of farms and pigs per farm were of 3,300 and 3,400, respectively. A recent Rabobank study (second quarter of 2023), however, concluded that the selling price of piglets should be of 68 € per head in order for the farmers to break even. A decrease in feed and energy cost is not expected for the near future, which will penalize the production of piglets for exports. For the last decades, The Netherlands has continued the depopulation of its pig heads, in an effort to lower agriculture environmental emissions, with increases in the subsidies and payments to farmers to close their farms. As a consequence, the decline in pigs slaughtered in the country from September 2023 to September 2022 was of 14% [15]. In 2018 pig density in The Netherlands was of 298 pigs/km^2^ [35].

On the other hand, farmers and technicians of the country are highly qualified, with a good expertise and rapid access to excellent technology, genetics, pig care, and nutrition, with emphasis in manure management practice. Sales of meat to other EU countries, such as Germany, Poland and Spain, together with exports to the UK by the main slaughterhouse and pork production chain of the country (close to 16.9 M pigs slaughtered in 2022) will facilitate, at a certain level, the maintenance of the pig business in the country. Altogether, the data make difficult to expect an increase in pig production in The Netherlands in the near future.

### France

France is the third larger producer of pig meat of the EU, with more than 70% of the pig herd concentrated in the three western regions of the country (Brittany with 56% of the pigs in 2020, Pays de Loire and Basse-Normandie). Traditionally, the main objective of pig production in France was to reach self-sufficiency with most farmers not dedicated exclusively to pig production [37]. The payments for early retirements, especially in the Western region of the country has reduced the inventory of pigs and sows in this period by 3.5% and 14.3%, respectively which makes difficult to meet the original objective. In fact, in spite of the protective strategy to limit the negative impact of current situation and the financial support to the sector by the government, the decline in pig production from 2000 to 2020 is close to 20% with a reduction of 80% in the number of farms (59,549 in 2000, 22,286 in 2010, and to 13,048 in 2020). The reduction has continued for the last three years and a further decrease in the numbers of medium/large size farm and pork production is expected for the next decade.

The evolution of the pig sector in France is moving towards specialized pig production, with little or not land and not necessarily related to a second activity. The importance of pig production, in terms of employment and economic interest, is decreasing and pig farming succession remains a major issue with over 30% of the pigs in the hands of farmers aged 55 years or older [37]. Consequently, based on the current situation, together with the potential risk of the spread of the ASF disease from Germany, makes difficult for the country to meet the original objective of auto-sufficiency. In favor of pig production in France is the low density of inhabitants (104.5 and 20.0 pigs/km^2^) respectively with one of the lowest pigs/inhabitant ratio within the key countries in Europe (Table 8).

### Germany

Germany was until recently the main pig meat producer in Europe but the census has suffered a continuous severe reduction for the last 10 years, a period (2012 to 2022) in which the number of pigs decreased by 2.8% (to 5.8 M pigs) and that of farms by 41% (to 12,000), with an increase in the number of pigs per farm from 929 to 1,248. The reasons for the decrease are multiple and include a reduction in production, with a sharp increase in cost, a reduction in consumption because of the increase in flexi-vegetarian population and a reduction of exports because of the ASF outbreaks.

From 2017 to 2022, the numbers of farms in Lower Saxony, the center of German pig meat production, decreased by 24.4% and in parallel, the pig population felt by 16% (from 8.7 to 7.4 M pigs). Historically, the production of pork meat reached a record high of 5.6 Mt in 2011 and a record low of 4.5 Mt in 2022. Moreover, the pig census in Germany continues decreasing with a figure census which is the lowest since German unification in 1990 (30.8 M pigs). According to preliminary data from Destatis [38], the pig inventory in Germany (May 3rd, 2023) stood at 20.7 M heads, down 7.3% (−1.62 M animals) from previous May 3rd, 2022. Compared to two years ago, the reduction was of 16.1% (−4.0 M animals). Moreover, the volume of pork produced in the first half of 2023 was of 2.1 M tons, a 9.4% decrease compared to the same period of the previous year [38]. To take into account that pork meat production in Germany includes the slaughter of pigs fattened in the country and pigs brought from other European states, primarily the Netherlands and Poland. In this respect, the number of slaughtered pigs of domestic origin decreased by 9.6% to just under 45.8 M animals whereas the number of imported pigs increased by 6.5% between years (over 1.2 M pigs in 2022). The total number of pigs slaughtered in the country in 2023 is expected to be 8% lower than in 2022 [15].

The Destatis [38] reported in May 2023, a 10.8% reduction in the number of pig farms (from 16,900 to 15,000) compared to same data in 2022. Moreover, the number of breeding sows declined by 6%, from 1.60 M in 2021 to 1.51 M one year later [20]. In fact, the drop in the number of sows has continued at a worrisome rate and the number of pigs slaughtered decreased by 9.2% from 2021 to 2022 and a further 2.2% decrease is expected for 2023 (Table 14). Because of problems related to the outbreaks of ASF virus in the wild boar population, the amount of pork exported from Germany in 2022, was of 1.5 Mt, a decrease of 20% compared to the past five years. Because of the high selling price and of cost of pork production, Germany imported 0.9 Mt of pork in 2022, originated mostly in other EU-27 countries. Some exports to other EU-27 countries, primarily Italy, Poland and The Netherlands compensate for the imports. On the other hand, pig meat consumption has decreased by 0.8% from 2022 to 2017 and a further decrease of 0.8% per year is expected in the near future. The German market, already struggling after the COVID-19 crisis and the ASF situation, is facing an economic crisis which is affecting not only to the farmers but also to the slaughterhouses and to the pig industry in general.

The reasons for the current situation in Germany, in line with those of other highly developed members of the EU, are multiple and include the following specific issues:

1) High incidence of ASF outbreaks (virus detected only in seven pig farms since 2020 until December 2022 but over 4,800 cases in wild boars) which impeded exports to China and other key Asian countries.
2) Increase in the flexitarian, vegetarian and vegan populations of the new generation, with a reduction in meat intake in favor of the concept “healthy way of life”. In this respect the average consumption in Germany for all kind of meats, although decreasing is of 59.7 kg/person.
3) Increase in regulatory pressure by the government for improvements in housing and husbandry conditions of the pigs and in pork quality [39]. In addition, the tight legislation in relation to farm size, environment control and animal welfare, which is increasing, makes difficult to think in a prompt recovery of the market.
4) Negative sentiments driven by the increases in animal feed and fuel cost and the ongoing risks associated with the spread of ASF, ending in a determinations of the profit margins.

As a summary, Germany is a candidate to show the sharpest decline in pork production of Europe. The reduction of the supply and the demand at the same time is causing a shrink age of the pork market which is not expected to recover in the near future.

### Spain

Spain is after China and the USA, the 3rd largest producer in the world. The white pig sector of the country is overwhelming intensive and heavily concentrated, with an ongoing process that began in the 1960s, based on the reduction to a minimum the number of pigs reared on backyards. The main period of increase in pig production started when the country became a member of the EU in 1986, and continued with the eradication of the ASF in 1995. At the end of this period, the pork meat self-sufficiency was of 110% and exports to the other members of the EU started. From 1999 to 2013, the number of pig units decreased by more than two thirds and in fact, 128,000 farms disappeared, while the number of pigs per farm quadruplicated. For the last 10 years (2014 to 2023) Spain has been the main responsible for the increase in pig census in the EU. The number of pig farms increased by 1.3% (from 47,374 to 48,021) with a slow decrease of 3% from 2023 to 2022. In the last 3 years (2023 to 2021) the number of pigs slaughtered decreased from 33.2 to 30.6 M and pork meat also decreased from 2.98 to 2.85 M tons [18]. Currently, the pig sector is an important part of the Spanish economy with the production of pork well managed by producers, industry, community and government.

The Spanish pork industry is very competitive and export oriented, in spite of the need to import most of the key ingredients (e.g., soybean meal and corn) used in feed formulas. Pork export increased yearly from 1.36 in 2011 to 3.09 Mt in 2021 of which, 1.2 Mt were destined to the EU market and 1.9 Mt to exterior markets [18,40]. In 2021/2022, Spain exported 56% of the total pork produced in the country and after the USA, is the 2nd larger exporter of the world. However, no further increases are expected because of the stabilization of the industry and a possible reduction of exports, particularly those to Asia. In relative terms, the swine sector of Spain has most of the advantages, indicated previously for the EU for optimal production and at the same time, suffers less the drawbacks than all the other members. The main competitive advantages of Spain, compared to other key countries are shown below.

**Figure 2:**
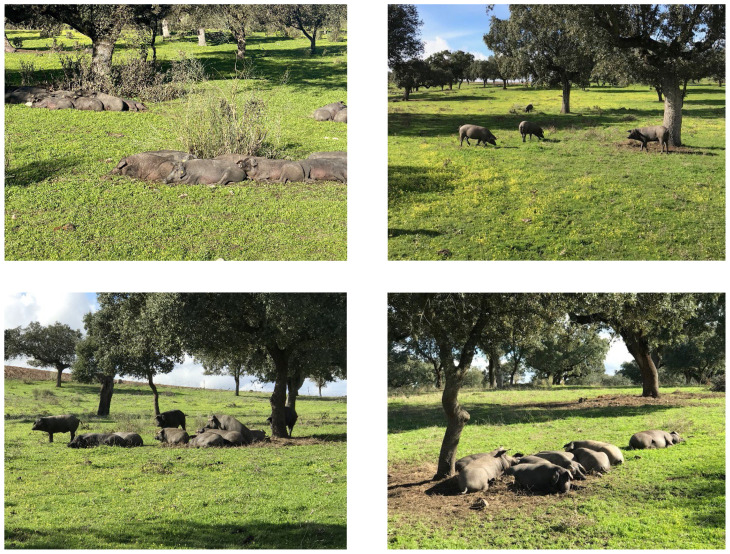
Iberian pig production (Spain), rearing under free range conditions.

1) Well develop, sound vertical integration system [41]. The Spanish pig market is characterized by its high degree of integration with most sites of production (feed mills, farms, veterinary services, slaughterhouses and packing facilities) owned by family groups with generational changeover (74% of all white pigs produced) or controlled (15%) by well-organized cooperatives [13]. Thus, only 11% of the slaughter pigs, is handled by independent free farmers, a number that has decreased continuously for the last 15 years. The high level of efficiency of the integrated systems (managed by members of the same family for the last 60 years) and the adequate size of the farms (most new farms with a capacity of over 1,000 sows or 600 fattening pigs) reduce substantially pork production cost.
2) Pork meat is an important sector of the Spanish economy, representing 50% of all the animal products. In addition, pork production creates work for the rural habitants of the depopulated areas, which reduces the pressure by consumers and local government legislation.
3) Spain is leader in Europe (and worldwide) in two key aspects: local consumption of pig meat and potential for pork exports. Self-sufficiency have increased from 122.1% in 2007 to 206.7% in 2022. In this period, apparent consumption per capita decreased from 62.3 kg to 51.4 kg but has remained constant for the last three years [40]. Even more, tourists visiting the country (e.g., expected to reach 84 M in 2023), consume high levels of dry-cured pork products, helping to the pig industry.
4) The density of the population in Spain is close to 94 habitants/km^2^ (48.0 M in total). However, if the urban population living in the big cities (e.g., Madrid, Barcelona, Sevilla, Valladolid, and Zaragoza) or in the cost (Atlantic, Mediterranean and Cantabric seas), which represent over 80% of the population, is not taken into consideration, the density of the rural population in the potential pig production areas, is below 22 habitants/km^2^, which reduces the competition for space between pigs and humans. In addition, most of the rural population lives in medium size villages, not necessary in the rural environment, which reduces further the competition problem. Even more, pig production is moving to the most depopulated areas of the interior of the country which reduces, in relative terms, the problems created by high density of farms and animals.
5) Wide diversification in pig production and pork systems (niche markets). Pig production in Europe is quite homogenous, with predominance of white pigs (crosses of Large White sows with Pietrain on synthetic high lean boars) slaughtered at 110 to 116 kg BW, previous male castration. The Spanish pig production system, however, is very diversified and highly more sophisticated, with a high percentage of pork products sold at added value prices. Details of the type and systems of pig meat production are presented below [13].
  • Standard lean white pigs: Based on Large White sows crossed with Pietrain or high muscular synthetic boars. Under this system pigs (70% of total production) are not castrated and slaughtered at around 110 kg BW, which reduces production cost and improve animal welfare. Hams, shoulders and loins are sold as fresh or as cured products, increasing products diversification and improving economic margins.
  • Heavy weight white pigs: Based on the crossing of Large White sows with Duroc boars (not necessarily high lean males). The pigs (18% of total production) are castrated and slaughtered at around 125 kg BW. Hams, shoulders and loins of these pigs are destined primarily to the dry-cured industry or sold as fresh, at high added value prices. Depending on the length and the characteristics of the dry-curing process, meat quality, cost of production and selling prices vary.
  • Young roasted piglets: Piglets (4% of total production) are slaughtered at weaning with seven to nine kg BW. The meat is destined to restaurants and to the HORECA distribution channel. The number of piglets sold through this channel is small in comparative terms but helps to reduce the offer when the price of pork from the traditional pigs is expected to decrease.
  • Iberian pigs: According to MAPA [18] the total census of Iberian pigs was of 3.4 M animals, in December 2022, with 0.33 M sows and 1.8 M heads over 50 kg BW. Iberian pigs (7% of total production) are the traditional pigs of Spain and until 1950, was the only source of fat and animal protein available for the peasants. Currently, Iberian pigs are an important part of the pork industry, of growing interest in the depopulated area of South-western Spain. The Iberian breed is one of the few examples of a domesticated pig which has adapted to a pastoral setting, provided that the land is reach in natural resources. The increase in the production of pigs under this type of extensive farming, is generating extra benefits because of the high quality of the final dry-cured products [13]. In contrast to the prevailing industrial, sophisticated, fully integrated model, the traditional agro-sylbo-pastural pig farming system based on the Iberian pig, with animals running freely and fed with grass, pasture and acorns (Quercus ilex fruits) are gaining interest, in spite of the high price. Because of the rearing of the pigs under free range conditions, the feeding based on the natural resources of the “Dehesa”, the type of meat produced (genetic, castration of males and females and 150 to 170 kg BW at slaughter) and the long dry-curing process (often over 24 months), the Iberian products reach in the market prices that might quadruplicate those of the white pigs. The main attributes that justify the preference of consumers for dry-cured Iberian products, are the taste, evocative flavor, marbling, intense meat color and muscle quality because of the exercise, high content in oleic acid and good image of the free-range production system (Figure 2). Information on the production system and type of Iberian pigs available in the market (Black, Red, Green, and White label, differing in nutrition, pig management, carcass quality characteristics and selling price), is shown in Table 9.
6) Adequate size of the pig complexes (farms dedicated to the production of Iberian pigs, smaller in size and organized for extensive production, are not included), similar to those for intensive production of Denmark and The Netherlands, making possible a reduction in production cost on the bases of the economy of scales [13,41,42].
7) Diversification of the offer. Pig products are consumed in Spain as fresh meat (8.58 kg per capita) or as transformed and dry-cured meat (10.45 kg per capita [18]). Fresh meat from white pigs is preferently channelled for exports. Dry-cured products are preferred to fresh meat cuts at the local market and are produced preferently, from all carcasses, especially from heavy white and Iberian pigs (over 80%). Recently, the offer of fresh meat from Iberian pigs, offered and sold at a higher price in the local HORECA market, is increasing.
8) Adequate cost of production, based on farm size, labor cost and mild climate conditions which reduces energy cost. Labor cost is lower than in other leader states, not only because of the relatively lower salaries in the depopulated areas of the country but also because of the design and size of the new facilities (frequently over 1,000 sows) which reduces cost per sow. In addition, the fattener units are built in depopulated areas with adequate size (over 600 fattening pigs per unit in new farms) with further reduction in cost. Moreover, the geography of the country and the need for organic matter, N and P of the land, in many cases never fertilized, reduces the impact of pig slurry on environmental contamination, improving the sustainability of the production system.
9) High standards for biosecurity, herd hygiene and pig management, which increases the acceptance of pig production by the consumers [43]. Current legislation regulates and improves the existing production systems, with limits on pig unit size in some areas, minimum distance between farms and villages and improving the management and the use of the excreta.
10) Good image of the Spanish pork production system all over the world. This good image, together with the benefits of “Spain”, a well-known country for tourism, increase the acceptance of the Spanish products in the export markets.

As per other EU countries, pork production in Spain has a series of drawbacks, as shown below.

• Reduced availability of traditional ingredients. Spain imports approximately 60% of the ingredients used in feed production, with soybean meal (95% of total use) and cereals (e.g., corn) under problems because of the political situation affecting Eastern Europe.
• New and tighter legislation for the control of farm management and nutrition practices aimed to improve animal welfare and environment sustainability, which in turn limits potential opening of new farms or any increase in density of pig production. The problem is limited in the depopulated areas of the centre of the country because the land in these areas needs extra fertilization to increase organic matter for cereal production. New measurements include changes in farm design and pig management with a ban to use in feeds ZnO, antibiotics and certain ingredients from deforested areas [44].
• Need to control outbreaks of key diseases such as PRRS, Lawsonia, circovirus and Streptococcus [43]. Biosecurity measures to control some diseases, such as ASF and tuberculosis, might need to improve in the case of the Iberian population, because of the extensive system of production and their interaction with other species (wild boars, deers, and other ruminant species). Spain has been free of any ASF outbreak for over 35 years but the problem might show up unexpectedly. The Spanish pork sector has to cope with the fight against this disease, which might affect future export opportunities [43].
• Implementation of new technologies to reduce feed cost, including better use of the ingredients and improved quality control of the feed manufacturing process. In the last 13 years, the number of feed mills in Spain has decreased by 20% (759 in 2023 vs 935 in 2009) [26]. In fact, in 2022, Spain had more feed mills than Germany, France, Netherlands, and Denmark together (759 vs 729). A further reduction in the number of feed mills, together with an increase in volume of the remaining mills, might be necessary in the future.
• Although meat consumption in Spain is high, there is a tendency to reduce it, mainly for health, sustainability and ethical reasons. Consequently, and increase in transparent information to the local population is needed [45].
• Geographical location. The Iberian Peninsula is placed in the southwest corner of Europe, close to the Maghreb countries. Consequently, pork exports possibilities are limited. However, the geographical location is today one of its main advantages, because distance (e.g., the sea) acts as a protection barrier against unwanted ASF outbreaks, affecting wild boars and domestic pigs in many European and African countries.

Consequently, based on all these considerations, a decrease in growth of the pork industry for the next years is expected. In fact, exports to Asian countries from July 2022 to July 2023 decreased as much as 41.8%, 31.5%, and 28.2% to Philippines, Taiwan, and South Korea, respectively. These decreases were compensated in part, by increased exports to Italy (23%) Romania (13%), Poland (12%) and other EU countries [18] but the reduction might be temporary and below those of other key EU countries. The halt expected might help to consolidate its leader position in the EU and worldwide in the long term. However, an increase in pork consumption and export, over current figures, are not easy to envisage.

**Figure 3:**
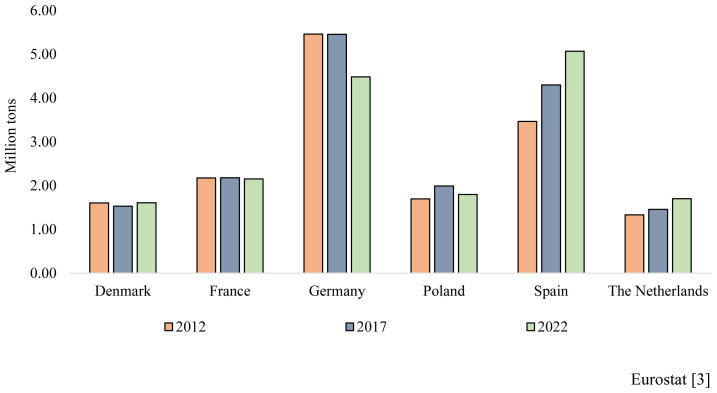
Pig meat production trends in key European Union-27.

As a summary, the analyses of the available information from main Agriculture Institutions [2] indicates that pig census and meat production in the EU will continue its current decline (Tables 7, 12, 13, and 14; Figure 3). The incidence of uncontrolled ASF, the distortion of the pork markets, the pressure of the legislation and the social insecurities of the rural population, together with the increase in flexitarian, vegetarian and vegan groups and the limited interest of the young generation of rural areas to work with pigs, complicate the future scenario of the sector in detriment of pig production. Moreover, the preference shown by the urban population for not having pigs “around” (e.g., second residences), complicate further the future of pig production in highly developed countries. In consequence, the pig sector will need a critical reorganization, moving animals from high-income, dense populated areas, with difficulties to handle and control contamination issues (e.g., Denmark, Germany, and The Netherlands) towards countries with extensive depopulated areas, plenty of space, with lower labor cost and reduced pressure against animal production by activists. Availability of local ingredients and easy access to ports will be needed. In addition, a further reduction of backyard production, an improvement in pig management, the implementation of new technologies and focusing on the production of added value pork products, with better margins than the traditional pork pieces or full carcasses, will be a must in the years to come.

## CONCLUSION

In summary, the current leading of the EU in the pig production and pork exports worldwide might be reduced in the next 10 years. The reasons are exposed in this document but real future trends will depend primarily on the control of the ASF and other pig diseases further legislation on meat quality, animal welfare and sustainability, not only in the European Union, but all over the world, will affect also future trends.

## Figures and Tables

**Table 1:** World meat market balance^1)^

Item	2018	2021	2022	2023^2)^	Change^2)^ (2023/2024)
Global production	-	356.9	362.6	363.9	+0.4
Bovine+ovine	86.6	91.3	93.0	92.9	−0.2
Poultry	123.2	138.2	140.8	142.7	+ 1.3
Pigs	120.7	120.9	122.3	121.7	−0.5
Trade		42.0	41.8	42.1	+0.6
Bovine+ovine	-	13.2	13.7	13.9	+1.2
Poultry	-	15.8	16.3	16.4	+1.0
Pigs	-	12.7	11.5	11.4	−1.0
Per capita consumption (kg/yr)	-	45.0	45.2	45.0	−0.4

1) Million tons (carcass weight equivalent).

2) Forecast.

OECD-FAO [2].

**Table 2:** Pork production worldwide^1)^ (Top countries summary)

Item	2012	2017	2021	2022	2023^2)^	2024^2)^
China	53.4	54.5	47.5	55.4	56.5	55.9
EU-27	22.5	23.7	23.6	22.3	21.8	21.1
USA	10.6	11.6	12.6	12.3	12.4	12.6
Brazil	3.3	3.7	4.4	4.4	4.4	4.8
Canada	1.8	2.0	2.1	2.1	2.0	2.0
Total world	108	112.1	107.9	114.5	115.5	115.4

1) Million tons (carcass weight equivalent).

2) Forecast (October, 2023).

USDA [4].

**Table 3:** Swine stocks worldwide1) (Main countries)

Item	2021	2022	2023^2)^
China	406	449	453
EU-27	146	142	134
USA	77	74	75
Brazil	37	36	34
Russia	26	26	26
Canada	14	14	14
Mexico	12	12	12
Total	750	784	778

1) Million heads.

2) Forecast (October, 2023).

USDA [4].

**Table 4:** Pork global trade worldwide^1)^ (Top countries summary)

Item	Year				% of total	
	
	2019	2022	2023^2)^	2024^3)^	2022	2023^2)^
Imports						
China	2,450	2,125	2,275	2,308	22	24
Japan	1,493	1,523	1,490	1,500	15	15
Mexico	985	1,299	1,310	1,300	13	14
UK	600	779	720	710	8	7
South Korea	694	735	720	705	7	7
Total	9,268	9,797	9,641	9,749	-	-
Exports						
EU-27	4,266	4,173	3,200	3,288	38	31
USA	2,867	2,878	3,067	3,152	26	30
Canada	1,286	1,415	1,310	1,305	13	13
Brazil	861	1,319	1,450	1,530	12	14
Total	10,382	10,940	10,144	10,365	-	-

1) Thousand tons (carcass weight equivalent).

2) Forecast (January, 2023).

3) Forecast (October, 2023).

USDA [4].

**Table 5:** European Union-27 export of selected pig products to extra European Union-27 countries, main destination^1)^

Item	Year				

	2002	2012	2020	2022^2)^	2023^3)^
China	54.9	579.7	3,337.5	1,550.1	865.8
United Kingdom	858.0	1,054.2	963.4	887.0	278.5
Japan	337.3	272.2	360.1	466.5	218.2
Philippines	12.9	73.2	152.9	428.2	671.3
South Korea	60.0	147.5	195.1	320.0	171.8
Extra EU	2,496.1	4,317.9	6,383.0	5,308.9	3,164.8

1) Thousand tons (carcass weight equivalent).

2) EU-27 exports by main country (% of total) in 2022: China (29.2).

United Kingdom (16.7), Japan (8.8), Philippines (8.1) and South Korea (6.0).

3) Data from January to September, 2023.

Eurostat [3].

**Table 6:** European Union-27 pork import from extra European Union-27 countries^1)^

Item	Year				

	2002	2012	2020	2022	2023^2)^
United Kingdom	92.5	187.5	177.0	140.0	71.3
Switzerland	2.41	18.8	19.3	19.2	17.1
Chile	3.88	7.70	2.32	4.9	8.3
Total	144.6	222.3	215.5	184.0	108.0

1) Thousand tons (carcass weight equivalent).

2) January to August, 2023.

Eurostat [3].

**Table 7:** Pig meat production^1)^ (Global trends)

Item	2022	2031	Change	

			Absolute	Relative (%)
Asia	63,917	71,492	+ 7,575	+ 11.9
Europe	31,266	29,573	−1,593	−5.1
North America	14,356	15,415	+ 1,059	+ 7.4
Latin America	9,159	9,814	+ 655	+ 7.2
Africa	1,634	1,955	+ 321	+ 19.6
Oceania	590	647	+ 57	+ 9.7

1) Thousand tons (carcass weight equivalent).

OECD-FAO [2].

**Table 8:** Demographic of the European Union-27 and pig census (November, 2022)

Country	Inhabitants (×10^6^)	Pigs (×10^6^)	Extension (10^6^ km^2^)	Density (per km^2^)		Ratio Pigs/people

				People	Pigs	
Spain	48.0	34.1	0.5048	93.9	67.5	0.72
Germany	84.4	21.4	0.3570	232.6	60.0	0.25
Denmark	5.9	11.5	0.0431	136.5	267.0	1.94
France	68.1	12.2	0.6435	104.5	20.0	0.18
Netherlands	17.8	10.7	0.0415	429.0	258.0	0.60
Poland	36.7	9.6	0.3130	121.0	30.7	0.26
Italy	58.8	8.7	0.3010	196.6	28.9	0.15
EU-27	448.4	134.3	4,080	109.6	32.9	0.30

Eurostat [3].

**Table 9:** Production of Iberian pigs (Classification and characteristics of the system)

Label^1)^	Genetics^2)^ (Iberian %)	Feeding^3)^	Slaughter age (months)	Carcass weight (kg)	Rearing density (m^2^)	Census (×10^3^)
Black	100	Acorns	14	115	104	425
Red	75	Acorns	14	108	104	55
Green	≥50	Compound feed^4)^	12	115	102	566
White	≥50	Compound feed	10	115	2	2,455

1) “Label classification” corresponds to type of feeding, management and quality of the meat.

2) Sows are Iberian breed in all cases.

3) Include pasture availability for the three first categories. Fattening period (100 to 160 kg BW) on free range conditions and fed on acorns and pasture exclusively.

4) Commercial feeds + cereals.

Higuera and others [13].

**Table 10:** Pigs slaughtered^1)^ at the European Union-27

Item	2012	2015	2021	Change^2)^ (%)
Spain	41.6	45.9	58.4	+ 27
Germany	58.2	59.3	51.8	−14
France	24.1	23.7	23.3	−1.7
Denmark	19.5	18.7	18.5	−1.1
Poland	19.2	21.2	21.1	0
Netherlands	14.3	15.5	17.2	+1.2
EU-27	246.6	246.7	249.5	+1.1

1) Million heads (June, 2022).

2) 2021/2015.

MAPA [18]; CESFAC [26]

**Table 11:** Pork production1 in the European Union-27 (Historical trend in exports and consumption per capita)

Item	2012^1)^	2019^1)^	2020^2)^	2021^2)^
Production^3)^	22.7	23.0	23.2	23.6
Exports^3)^	2.2	4.2	4.9	4.7
Consumption^4)^ (kg per capita)	32	33	32	33
Self-sufficiency (%)	111	121	126	125

1) EU-28 (July, 2022).

2) EU-27 (July, 2022).

3) Million tons (carcass weight equivalent), UK included.

4) Carcass weight equivalent.

CESFAC [26].

**Table 12:** Pig inventory^1)^ in the European Union-27

Item	Total stocks				

	2016	2020	2021	2022^2)^	Change^3)^ (%)
Spain	29.3	32.8	34.4	34.1	−0.9
Germany	27.4	26.9	23.8	21.4	−10.2
Denmark	12.3	13.4	13.2	11.5	−12.2
France	12.8	13.4	12.9	12.2	−5.9
Netherlands	11.9	11.5	10.9	10.7	−1.5
Poland	11.1	11.7	10.2	9.6	−6.0
Italy	8.5	8.5	8.5	8.7	+ 2.3
EU-27	142.6	145.9	141.7	134.3	−5.2

1) Million heads.

2) MAPA [18] data, December 2022.

3) 2022/2021.

MAPA 2023; Eurostat [3].

**Table 13:** Pig meat production^1)^ in the European Union-27

Item	2017	2021	2022	Change (%)
Spain	4.30	5.18	5.07	−2.2
Germany	5.45	4.95	4.49	−9.8
France	2.17	2.20	2.15	−2.3
The Netherlands	1.46	1.72	1.68	−2.4
Denmark	1.53	1.72	1.61	−6.4
Poland	1.99	1.98	1.79	−9.6
Italy	1.47	1.33	1.24	+ 6.0
EU-27	22.5	23.4	22.1	−5.6

1) Million tons (carcass weight equivalent). March, 2023.

Eurostat [3].

**Table 14:** Breeding sow census^1)^ in selected countries of the European Union-27

Item	December 2021	June 2022	June 2023	Change^2)^ (%)
Spain	2,712	2,699	2,725	+ 1.9
Germany	1,602	1,510	1,394	−6.7
France	941	917	879	−3.2
Denmark	1,245	1,201	1,123	−5.7
Netherlands	918	925	882	−4.1
Poland	665	617	591	−2.6
Total^3)^	10,092	9,809	9,483	−2.2

1) Million heads (December 2022 and 2021).

2) Variation from June 2023 to 2022.

3) Selected member States (n = 13).

Eurostat [3].

**Table 15:** Feed compound production^1)^, worldwide

Item	2021 (×10^9^ Mt)	2022 (×10^9^ Mt)	Growth (%)
Asia pacific	468	465	−0.5
Europe	276	263	−4.7
North America	259	261	+ 0.9
Latin America	188	191	+ 1.6
Total	1,272	1,266	−0.4

1) ×10^9^ tons.

Alltech [25].

**Table 16:** Worldwide feed compound production (Key countries)^1)^

Item	2021	2022	Growth (%)
China	268	261	−2.8
USA	238	240	+1.0
Brazil	81	82	+0.9
India	44	43	−1.6
Mexico	40	40	+0.9
Russia	33	34	+3.5
Spain	36	31	−12.8
Germany	25	24	−0.4
Total	812	809	−0.4

1) Million tons, industrial.

Alltech [25].

**Table 17:** Industrial feed compound production^1)^ in the European Union-27

Item	Swine			Poultry	Bovines	Total feeds (EU-27)	
			
	2021	2022^2)^	2023^3)^	2022	2022	2022	2023
Spain	13.4	12.3	12.2	4.3	8.9	25.8	25.6
Germany	9.4	8.5	8.1	6.2	6.2	21.9	21.3
France	4.8	4.5	4.4	7.7	5.2	19.2	19.3
Netherlands	4.8	4.7	4.5	4.1	4.3	14.3	14.0
Denmark	2.9	2.6	2.3	0.7	1.3	4.7	4.0
EU-27	52.7	49.2	47.6	47.2	40.9	146	144

1) Million tons.

2) Estimated.

3) Forecast.

CESFAC [26].

**Table 18:** Ingredients used in compound feeds^1)^ in the European Union-27 (Historical data)

Item	2011	2019	2021
Cereals	72.2	83.0	76.5
Oil seed meals	40.8	40.8	37.2
Food products	17.4	20.1	18.0
Protein grains	1.9	2.2	2.0
Fats and oils	2.7	2.8	2.6
Total	149	165	150

1) Million tons, industrial.

CESFAC [26].

**Table 19:** Volume of oilseed meals used in industrial compound feed^1)^ (European Union-27)

Item	Soybean	Rapes	Sunflower	Palm kernel	Total
Spain	5.3	0.59	0.77	0.12	6.85
Germany	3.0	4.12	0.38	0.18	7.75
France	3.4	2.50	1.30	0.12	7.49
Netherlands	3.2	0.80	0.21	0.90	5.17
Denmark	1.5	0.69	0.19	0.07	2.42
Total	28.3	12.9	3.0	1.4	50.1

1) Million tons, 2021.

CESFAC [26].

**Table 20:** Consumption of oilseed meals^1)^ (European Union-27)

Ingredient	% of total	Amounts imported^2)^ (Mt)	

		EU-27	Spain
Soybean meal	56	16.4	2.78
Rape seed meal	26	0.58	0.55
Sunflower meal	12	2.35	0.17
Palm kernel meal	3	1.44	0.13
Total oilseed meal	100	21.0	3.66

1) Percentage.

2) Million tons, industrial.

AFOEX-FEDOIL [28].
